# Protective effect of various toothpastes and mouthwashes against erosive and abrasive challenge on eroded dentin: an in vitro study

**DOI:** 10.1038/s41598-024-59631-1

**Published:** 2024-04-24

**Authors:** Mahtab Memarpour, Saba Jafari, Azade Rafiee, Marzieh Alizadeh, Mehrdad Vossoughi

**Affiliations:** 1 Oral and Dental Disease Research Center, Department of Pediatric Dentistry, School of Dentistry, Shiraz University of Medical Sciences, Shiraz, Iran; 2 Student Research Committee, Department of Pediatric Dentistry, School of Dentistry, Shiraz University of Medical Sciences, Shiraz, Iran; 3 Oral and Dental Disease Research Center, School of Dentistry, Shiraz University of Medical Sciences, Shiraz, Iran; 4 Mental Health Research Center, Psychological Health Research Institute (PHPRI), Iran University of Medical Sciences, Tehran, Iran

**Keywords:** Casein phosphopeptide-amorphous calcium phosphate, Dentin, Erosion, Fluoride, Remineralization, Nanohydroxyapatite, Stannous, Medical research, Preclinical research

## Abstract

The study aimed to compare various toothpastes and mouthwashes on permanent tooth dentin after erosive and abrasive challenges. 130 sound premolars dentin were randomly submitted to an initial erosive challenge and a cycle of erosive and abrasive challenges for five days. The five experimental groups (n = 26) were: (1) Control group (artificial saliva), (2) Elmex erosion protection toothpaste and mouthwash, (3) Vitis anticaries biorepair toothpaste and mouthwash, (4) Oral B Pro-expert toothpaste and Oral B Fluorinse mouthwash, and (5) MI Paste ONE toothpaste and Caphosol mouthwash. Microhardness, surface roughness values, and the topographical characteristics of the dentin surface were assessed. The highest percentage of recovered dentin microhardness (%RDMH) value was observed in groups 2 and 4, followed by groups 5 and 3, respectively. The %RDMH values in groups 2 and 4 did not demonstrate a significant difference (p = 0.855). The highest percentage of improvement in surface roughness was recorded in groups 2 and 4, with no significant differences (p = 0.989). The atomic force microscopy (AFM) findings were consistent with the surface roughness data. The best recovery of dentin microhardness and roughness were measured with the Elmex and Oral B toothpaste and mouthwash, followed by MI Paste ONE toothpaste and Caphosol mouthwash and Vitis anticaries biorepair toothpaste and mouthwash.

## Introduction

Tooth erosion is defined as the pathological breakdown of dental hard tissue (enamel, dentin) resulting from chemical acid exposures unrelated to bacterial biofilm^1^. Dentin represents a more complex erosion process than tooth enamel due to its higher content in the organic matrix. The erosion process in dentin initiates from the peritubular dentin with a higher mineral content compared to intertubular dentin. Subsequently, the dissolution of hydroxyapatite crystals between the dentinal tubules leads to exposure of the organic collagen matrix fibers. The changes in the dentin fluid flow may cause tooth hypersensitivity and pain^2,3^. Unlike the demineralization processes occurring under the plaque with a relatively constant environment, the erosion process is affected by many factors, such as pH, buffer capacity, the degree of calcium (Ca) and phosphate (P) saturation, and the absence or presence of erosion inhibitors like fluoride^4,5^. Due to a rather constant concentration of Ca and P in the plaque fluid during the caries process, a critical pH value of around 5.5 can be measured. However, the higher concentrations of Ca and P in the solution adjacent to tooth mineral compared to the plaque fluid result in tooth mineral dissolution protection even at lower pH values as the "critical" pH for caries^6^. In other words, erosion has no defined critical pH as the caries process does^5^. Various factors should be considered for dental erosion prevention, including nutrition and patient-related factors (such as oral hygiene, history of dental treatments, occupation, saliva, etc.). To prevent the lesion progression by removing the risk factors and implementing the appropriate intervention, it is crucial to identify the initial signs of tooth erosion^7^. Abrasion of eroded surfaces occurs as a result of friction of abrasive materials against the tooth surface. The use of a abrasive toothpaste, hard bristles of toothbrush, vigorous brushing force, and consumption of abrasive foods can cause such tooth surface loss^8^.

Various compounds, including fluoride^3,7,9–11^, casein phosphopeptide-amorphous calcium phosphate (CPP-ACP)^11–14^, nanohydroxyapatite (nHA)^11,14–16^, chitosan^17,18^, and protease inhibitors^7,19^ have been used in the forms of toothpaste, mouth rinse, gel, and varnish to prevent tooth erosion progression and alleviate hypersensitivity. Different forms of fluoride, including sodium fluoride (NaF), amine fluoride (AmF), monofluorophosphate (MFP), and stannous fluoride (SnF_2_) with different concentrations and pH levels, have demonstrated a significant reduction in enamel dissolution and an increase in resistance to acid attacks by deposition of protective calcium fluoride layer on the tooth surface^9^. Studies comparing the effects of NaF with a commercially available product (Elmex, GABA) containing stannous chloride (SnCl_2_), NaF, and AmF on enamel and dentin erosion have shown conflicting results^3,10^. CPP-ACP facilitates the remineralization of eroded surfaces by increasing the levels of Ca and P in dental plaque and prevents further enamel/dentin demineralization by its buffering properties at pH drops during erosive challenges^13,20^. In another study, morphological and chemical evaluation of NaF varnish, desensitizing cream containing NaF and 20% nHA, toothpaste with NaF and tricalcium phosphate, and toothpaste containing NaF and CPP-ACP (CPP-ACPF) on eroded root dentin was evaluated^11^. The results showed that the fluoride varnish and CPP-ACPF toothpaste were effective in preventing morphological alterations, and they were the only materials that demonstrated an increase in the Ca and P content after treatment^11^. Hydroxyapatite (HA), the primary mineral constituent of enamel, dentin, and bone, is a bioactive and biocompatible compound. It can penetrate deep into the eroded dentin and lead to remineralization of the surface and block of dentinal tubules^9,17^. However, its independent protective effect against erosion is not yet clearly defined^15^. Hydroxyapatite-containing toothpaste showed the lowest percentage of remineralization and protection effect against subsequent demineralization compared to AmF-containing toothpaste and fluoride- and hydroxyapatite-free toothpastes^16^. nHA also showed the least remineralizing properties compared to CPP-ACPF and bioactive glass on eroded enamel lesions^14^.

Considering the rising prevalence of dental erosion in children and adolescents due to increased consumption of acidic drinks, identifying the effective combination of toothpaste and mouthwash for the prevention of erosion and remineralization of eroded tooth surfaces is valuable. Hence, the aim of this in vitro study was to investigate the effect of different combinations of toothpastes and mouthwashes on the erosion/abrasion of permanent tooth dentin.

## Methods and materials

### Study design

A total of 143 sound premolars, extracted for orthodontic reasons from individuals aged 18–20, were collected following the approved protocol of the Ethics Review Committee of Shiraz University of Medical Sciences (IR.SUMS.DENTAL.REC.1400.146). Extracted teeth were thoroughly cleaned from any remaining tissues and debris using a prophylaxis brush and were disinfected by immersion in 0.1% chloramine T solution for four weeks. The samples were stored in deionized water that was replaced on a weekly basis at 25 °C until use. Before the onset of the study, the samples underwent examination under a stereomicroscope (Motic K, Wetzlar, Germany) at × 40 magnification to exclude teeth with defects or fracture lines. In total, 130 teeth fulfilled the inclusion criteria. Prepared samples were subjected to erosive and abrasive challenges and various combinations of toothpastes and mouthwashes. The surface microhardness, surface roughness values, and dentin topography were assessed at three stages: sound dentin (baseline), dentin with initial erosion, and remineralized dentin.

### Sample preparation

The roots were removed about 2 mm below the cemento-enamel junction, and the crowns were inserted in self-curing acrylic resin, aligning the buccal surface parallel to the mold. The enamel was precisely removed using a high-speed handpiece, and the exposed dentin was polished with 320-, 600-, 1200-, and 2400-grit waterproof silicon carbide papers (Buehler, Lake Bluff, IL, USA). The polished samples were then immersed and cleaned in an ultrasonic bath for 5 min. The complete removal of the enamel from the tooth surface was justified with the stereomicroscope at × 10 magnification. Two layers of nail varnish covered the dentin surface with an exposed 2 × 5 mm window to standardize the intervention surface. Then, the samples were randomly assigned to five experimental groups. Each group comprised 26 teeth, with 12 teeth allocated for surface microhardness testing, 12 teeth for surface roughness measurement, and two teeth for atomic force microscopy (AFM) evaluation.

### Initial erosive lesion creation and pellicle formation

To induce the initial erosive lesion, each sample was exposed to 20 mL of 0.1% citric acid solution with a pH of 2.5 for 30 min at 25 °C on a stirrer (Alfa D500, Iran) at 60 rpm, with the citric acid being refreshed every 5 min. Next, the samples were subjected to a 30-s rinse using deionized water.

To better simulate the oral cavity conditions, the samples were stored in artificial saliva (20 mL each) at 25 °C for an hour to create salivary pellicle on the dentin surfaces. The artificial saliva composition consisted of 0.2 mM glucose, 0.1 mM C_8_H_15_NaO, 9.9 mM NaCl, 1.5 mM CaCl_2_·H_2_O, 3 mM NH_4_Cl, 17 mM KCl, 2 mM NaSCN, 2.4 mM K_2_HPO_4_, 3.3 mM urea, 2.4 mM NaH_2_PO_4_, and 11 µM ascorbic acid (pH 6.8)^21^. The artificial saliva required for pellicle formation, erosive challenge, and toothpaste slurry preparation was prepared weekly and stored at 25 °C.

### Group allocation

The five experimental groups were as follows:

Group 1 (negative control):

The samples were immersed in artificial saliva during erosive and abrasive challenges.

Group 2 (positive control) (toothpaste and mouthwash containing AmF (olaflur), NaF, and SnCl_2_:

The samples were exposed to Elmex erosion protection toothpaste, containing 1400 ppm F^−^ and 3500 ppm Sn^2+^ (GABA International AG, Switzerland), followed by Elmex erosion protection mouthwash, containing 500 ppm F^−^ and 800 ppm Sn^2+^.

Group 3 (toothpaste and mouthwash containing sodium monofluorophosphate (SMFP) and hydroxyapatite):

The samples were subjected to Vitis anticaries biorepair toothpaste, containing 1450 ppm F^−^ and 4500 ppm hydroxyapatite (VITIS Oral Health C/O Dent-O-Care, UK), followed by rinsing with Vitis anticaries biorepair mouthwash, with 226 ppm F^−^ and 125 ppm hydroxyapatite.

Group 4 (toothpaste with NaF and SnF_2_ and mouthwash with NaF):

Oral B Pro-expert toothpaste (1450 ppm F^−^ and 3230 ppm Sn^2+^) (Procter and Gamble, USA) was used, followed by rinsing with Oral B Fluorinse mouthwash (226 ppm F^−^).

Group 5 (toothpaste with NaF and CPP-ACP and mouthwash with supersaturated Ca and P ions):

Intervention on the erosive surface was performed using MI Paste ONE toothpaste (1100 ppm F^−^) (GC America), followed by rinsing with Caphosol mouthwash (EU Pharma, USA), containing two vials A and B, with an equal mixing ratio.

The toothpastes and mouthwashes used in the present study and their compositions are demonstrated in Table 1.

**Table 1:** The composition of toothpastes and mouthwashes used in the present study.

Toothpaste/mouthwash	Composition	Manufacturer
Elmex erosion protection toothpaste	Aqua, Glycerin, Sorbitol, Hydrated Silica, Hydroxyethylcellulose, Aroma, Cocamidopropyl Betaine, CI 77891, Sodium Gluconate, Stannous Chloride (3500 ppm Sn^+^), Alumina, Chitosan (0.5%), Sodium saccharin, Sucralose, Cinnamal, Limonene, Sodium Fluoride and Olaflur (1400 ppm F^−^)	GABA International AG, Therwil Switzerland
Elmex enamel professional mouthwash	Aqua, Glycerin, Sodium Gluconate, PEG-40 Hydrogenated Castor Oil, Olaflur, Aroma, Stannous Chloride (800 ppm Sn^2+^), Sodium Fluoride (500 ppm F^−^), Cocamidopropyl Betaine, Sodium Saccharin	GABA International AG, Therwil Switzerland
Vitis anticaries with nonorepair toothpaste	Aqua, Glycerin, Sorbitol, Hydrated Silica, Xylitol, Titanium Dioxide, Sodium Monofluorophosphate (1450 ppm F^−^), Sodium Lauryl Sulfate, Xanthan Gum, PEG-40, Hydrogenated Castor Oil, Hydroxyapatite (0.45%, 4500 ppm), Menthone Glycerin acetal, Sodium Saccharin, Citric Acid, Sodium Methylparaben, Potassium Acesulfame, Aroma	VITIS Oral Health C/O Dent-O-Care, London, UK
Vitis anticaries mouthwash	Aqua, Propylene Glycol, Glycerin, Xylitol, PVP, PEG-40 Hydrogenated Castor Oil, , Sodium Hexametaphosphate, Sodium Benzoate, Sodium Monofluorophosphate (226 ppm F^−^), Carbomer, Sodium Lauryl Sulfate, Sodium Methylparaben, Potassium Acesulfame, Hydroxyapatite (0.0125%, 125 ppm), Menthone Glycerin acetal, Sodium Saccharin, Aroma, CI 19,140, CI 42,051	VITIS Oral Health C/O Dent-O-Care, London, UK
Oral B Pro-expert toothpaste	Glycerin, Hydrated Silica, Sodium Hexametaphosphate, Propylene Glycol, PEG-6, Aqua, Zinc Lactate, Sodium Lauryl Sulfate, Aroma, Sodium Gluconate, Chondrus Crispus Powder, Trisodium Phosphate, Stannous Fluoride (1100 ppm F^−^) (3230 ppm Sn^+2^), Sodium Saccharin, Xanthan Gum, Copernicia Cerifera Cera, Cinnamal, Silica, Sodium Fluoride (350 ppm F^−^)	Procter & Gamble, Germany
Oral B Fluorinse mouthwash	Aqua, Sorbitol, Xylitol, Alcohol, PEG-40, Hydrogenated Castor, Oil, Methylparaben, Aroma, Sodium hydroxide, Sodium Saccharin, Cinnamal, Propylparaben, Eugenol, CI 42090, Sodium Fluoride (226 ppm F^−^)	Procter & Gamble, Germany
MI Paste ONE	Sodium fluoride (1100 ppm F^−^), Potassium nitrate, Pure water, Glycerol, RECALDENT(CPP-ACP), Sorbitol, CMC-Na, Propylene glycol, Silicon Dioxide, Titanium Dioxide, Xylitol, Phosphoric acid, flavoring, Methyl Salicylate, Sodium Saccharin, Ethyl p-hydroxybenzoate, Propyl p-hydroxybenzoate, Butyl p-hydroxybenzoate, Sodium-N-lauryl sarcosinate	GC America
Caphosol A and B mouthwash	Dibasic Sodium Phosphate 0.032, Monobasic Sodium Phosphate 0.009, Calcium Chloride 0.052, Sodium Chloride 0.569, Purified Water	EU Pharma, USA

### Erosive challenge protocol

The erosive challenge protocol consisted of four times a day (10 AM, 12 PM, 2 PM, and 4 PM) exposure to 0.1% citric acid solution at pH 2.5 for 90 s over five days. Following each challenge, the samples were rinsed with deionized water for 10 s and stored in 20 mL of artificial saliva while incubated at 25 °C until the next challenge. Besides, the samples were kept in artificial saliva overnight. Toothpaste and mouthwash of each group were applied on the dentin surfaces 30 min after the first and the last acid exposure. Citric acid and artificial saliva were replaced daily.

### Remineralization of the eroded dentin surface

Toothpaste slurry for groups 2 to 5 was prepared daily by mixing 100 mg of toothpaste with 300 mg of artificial saliva using a stirrer device at 60 rpm. The abrasive challenge was performed using a two-chamber automatic toothbrushing device (Shiraz Electric, Shiraz, Iran). After placing the samples inside the chambers filled with toothpaste slurry for 1 min, the dentin surfaces were brushed with a soft toothbrush (Oral B Health Clean Manual Toothbrush, Procter and Gamble, USA) under a constant force of 2.5 N with 120 strokes per minute for 15 s. Each tooth was then rinsed with deionized water for 10 s and immersed in mouthwash for 30 s at 60 rpm to imitate mouthwash rinsing in the oral cavity. The samples in group 1 (negative control) were brushed with artificial saliva without using toothpaste and mouthwash. At the end of the fifth day, laboratory tests were carried out to evaluate the changes in the surface microhardness, roughness, and dentin topography.

### Surface microhardness test

The microhardness values of each group (n = 12) were measured using a Vickers diamond indenter device (ZwickRoell, Fürstenfeld, Austria) at 50 g force for 15 s and at five points of 50 µm distance per sample. The measurements were repeated at three stages, including sound dentin (baseline), dentin with initial erosion, and remineralized dentin. The percentage recovery of dentin microhardness (%RDMH) was calculated using the average Vickers hardness number (VHN) at each group with the following formula:

$$\mathrm{\%RDMH}=\frac{\mathrm{ VHN\,of\,remineralized\,dentin }-\mathrm{VHN\,of\,eroded\,dentin }}{\mathrm{VHN\,of\,sound\,dentin }-\mathrm{VHN\,of\,eroded\,dentin }}\times 100$$

### Surface roughness test

To assess the surface roughness (Ra) of the samples in each group (n = 12), a contact profilometer (TESA RUGOSURF 20, Switzerland) was used to scan five points with an equal distance of 250 µm. A silicone putty jig, providing an equal area of exposed dentin, was constructed on each mold to allow for multiple measurements. The Ra values corresponding to sound dentin (baseline), dentin with initial erosion, and remineralized dentin were recorded and analyzed with RUGOSOFT software.

The percentage recovery of dentin roughness (%RDR) was calculated as follows:

$$\mathrm{\%RDR}=\frac{\mathrm{ Ra\,of\,remineralized\,dentin }-\mathrm{Ra\,of\,eroded\,dentin }}{\mathrm{Ra\,of\,sound\,dentin }-\mathrm{Ra\,of\,eroded\,dentin }}\times 100$$

Furthermore, the percentage of surface loss of the remineralized dentin compared to eroded and sound dentin (baseline) was calculated with the following equations:

$${\mathrm{\%Surface\,loss}}_{{\text{remineralization}}-{\text{erosion}}}=\frac{\mathrm{ Ra\,of\,remineralized\,dentin }-\mathrm{Ra\,of\,eroded\,dentin }}{\mathrm{ Ra\,of\,eroded\,dentin }}\times 100$$

$${\mathrm{\%Surface\,loss}}_{{\text{remineralization}}-{\text{baseline}}}=\frac{\mathrm{ Ra\,of\,remineralized\,dentin }-\mathrm{Ra\,of\,sound\,dentin }}{\mathrm{ Ra\,of\,sound\,dentin }}\times 100$$

### Surface topographic evaluation

Two samples in each group were randomly selected for AFM evaluation (n = 10). The surface characteristics of the dentin samples were examined utilizing an AFM device (AFM, JPK Nanowizard II apparatus, JPK instruments, Berlin, Germany) in conjunction with a nonconductive silicon nitrite cantilever (Acta-Probe, APPNano, CA) and a piezoelectric scanner.

The area of measurements for each sample was 5 µm × 5 µm.

### Statistical analysis

The data analysis was conducted using SPSS version 22 software (IBM, NY, USA). The comparison of surface microhardness and roughness values in the five different groups was performed using one-way ANOVA and Tukey HSD post hoc tests. The %Surface loss _remineralization-baseline_ among five groups were compared by one-way ANOVA and Tukey HSD post hoc test. The remineralization column, ∆ values between the initial erosion (time point two) and the remineralization (time point three) (∆_32_), %RDMH, %RDR, and %Surface loss_Remineralization-erosion_ among five groups were compared by one-way ANOVA and Tamhane's T2 post hoc test. Also, repeated measure ANOVA and Sidak post hoc test were used for intra-group comparisons of sound dentin, eroded dentin, and remineralized dentin microhardness and roughness values. A significant level of 0.05 was considered for all statistical tests.

### Ethics approval

The study was approved by the Ethics Review Committee of the School of Dentistry, Shiraz University of Medical Sciences (IR.SUMS.DENTAL.REC.1400.146).

### Consent to participate

All methods were performed in accordance with the relevant guidelines and regulations (Declaration of Helsinki). Written informed consents for the use of the teeth were obtained from the participants.

## Results

### Surface microhardness

Table 2 demonstrates the mean ± SD values of surface microhardness of sound, eroded, and remineralized dentin, as well as %RDMH in each group. At the baseline (sound dentin), the VHN values ranged from 40.54—55.77 (mean ± SD: 47.39 ± 4.22 VHN), with no statistically significant differences between groups (p = 0.769). The erosive challenge significantly reduced the surface microhardness values of all samples compared to the sound dentin (p < 0.001), which varied between 37.22—30.46 VHN (mean ± SD: 30.29 ± 3.8 VHN). However, no inter-group significant difference was found (p = 0.142). Except for the negative control group, the VHN values of groups 2 to 5 significantly increased compared to the eroded dentin surface after exposure to the combination of toothpaste and mouthwash (p < 0.001, all). Exposure to the toothpaste slurry and the mouthwash significantly increased the dentin microhardness in groups 2 (Elmex), 4 (Oral B) and 5 (MI Paste ONE/Caphosol) compared to the control group (p = 048, p < 0.001, and p = 0.015, respectively). The comparison of the ∆ values between the erosion stage (time point two) and the remineralization stage (time point three) (∆_32_) demonstrated a significant difference between the two time points (p < 0.001), which is presented in Table 3. To better compare the dentin microhardness values at various stages, the %RDMH was also calculated using the previously explained formula. The highest %RDMH was observed in groups 2 (Elmex) and 4 (Oral B), with no significant difference (p = 0.855). Groups 5 (MI Paste ONE/Caphosol) and 3 (Vitis) showed a significant difference (p = 0.023). Group 1 showed a negative %RDMH value, as it was not exposed to toothpaste slurry and mouthwash (mean ± SD: − 3.05 ± 0.79 VHN).

**Table 2:** The dentin surface microhardness values (mean ± SD) and the percentage recovery of dentin microhardness (%RDMH) among the study groups (n = 12).

Group	Stage			
	Sound dentin	Initial erosion	Remineralization	%RDMH
(1) Negative control	47.93 ± 4.13 ^a,A^	29.38 ± 2.87^b,A^	28.88 ± 2.98^b,A^	− 3.05 ± 0.79 ^A^
(2) Elmex TP and MW	46.31 ± 4.14^a,A^	29.17 ± 4.79^b,A^	33.44 ± 4.55^c,B^	25.05 ± 2.44 ^B^
(3) Vitis TP and MW	48.29 ± 3.23^a,A^	29.18 ± 2.00^b,A^	31.67 ± 1.97^c,AC^	13.14 ± 2.12 ^D^
(4) Oral B TP and MW	47.69 ± 4.67^a,A^	31.76 ± 3.70^b,A^	35.46 ± 3.25^c,B^	23.58 ± 2.70 ^B^
(5) MI paste ONE TP and Caphosol MW	46.73 ± 5.10^a,A^	31.94 ± 4.43^b,A^	34.26 ± 4.23^c,BC^	15.93 ± 1.83 ^C^

*TP* toothpaste, *MW* mouthwash.

In each row, means with the same lowercase letter are not significantly different (Intragroup analysis).

In each column, means with the same capital letter are not significantly different (intergroup analysis).

**Table 3:** The difference between the initial erosion stage (time point two) and the remineralization stage (time point three) for both the hardness and the roughness values.

Group	∆_32_ of hardness values	∆_32_ of roughness values
(1) Negative control	− 0.55 ± 0.14^A^	0.02 ± 0.01^A^
(2) Elmex TP and MW	4.27 ± 0.65^B^	− 0.22 ± 0.03^B^
(3) Vitis TP and MW	2.49 ± 0.40^C^	− 0.14 ± 0.03^C^
(4) Oral B TP and MW	3.70 ± 0.98^B^	− 0.21 ± 0.04^B^
(5) MI paste ONE TP and Caphosol MW	2.31 ± 0.47^C^	− 0.15 ± 0.03^C^
p value	p < 0.001	p < 0.001

In each column, means with the same capital letter are not significantly different (intergroup analysis).

∆32: The difference between the erosion stage (time point two) and the remineralization stage (time point three).

### Surface roughness

Analysis of the profilometry data of the sound dentin (baseline) demonstrated the surface roughness of 0.22 to 0.36 nm (mean ± SD: 0.3 ± 0.03 nm), and no significant difference was observed between the groups (p = 0.733). The surface roughness of the eroded dentin surface in all groups increased dramatically compared to the baseline (p < 0.001, all), which ranged from 0.54 to 0.67 (mean ± SD: 0.61 ± 0.03 nm). The surface roughness values among groups were non-significant after the initial erosion (p = 0.503). Remineralization with toothpaste and mouthwash combination decreased the surface roughness values in all experimental groups (groups 2 to 5), with p < 0.001 for all groups. The difference in the remineralization values between groups 2 (Elmex) and 4 (Oral B) (p = 0.064), as well as groups 3 (Vitis) and 5 (MI Paste ONE/Caphosol) (p = 0.079), were non-significant. The comparison of the ∆ values between the erosion stage (time point two) and the remineralization stage (time point three) (∆_32_) demonstrated a significant difference between the two time points (p < 0.001), which is presented in Table 3.

Group 2 (Elmex) (72.40 ± 9.84 nm) and group 4 (Oral B) (68.41 ± 11.06 nm) revealed the highest %RDR, followed by group 5 (MI Paste ONE/Caphosol) (47.83 ± 8.47 nm) and group 3 (Vitis) (44.8 ± 9.22 nm), respectively. The difference in the %RDR values between groups 2 and 4 (p = 0.989) as well as groups 3 and 5 (p = 0.995) were non-significant. Table 4 demonstrates the dentin surface roughness (mean ± SD) and the %RDR values among the groups.

**Table 4:** The dentin surface roughness (mean ± SD), the percentage recovery of dentin roughness (%RDR) values, and the percentage of surface loss of the remineralized dentin compared to eroded and sound dentin values among the groups (n = 12).

Group		Stage				
	Sound dentin	Initial erosion	Remineralization	%RDR	%Surface loss_intervention-erosion_	%Surface loss_remineralization-baseline_
(1) Negative control	0.30 ± 0.04^a,A^	0.62 ± 0.03^b,A^	0.64 ± 0.03^b,A^	− 5.51 ± 1.18 ^A^	2.82 ± 0.66 ^A^	113.49 ± 25.06 ^A^
(2) Elmex TP and MW	0.29 ± 0.03^a,A^	0.60 ± 0.03^b,A^	0.38 ± 0.01^c,B^	72.4 ± 9.84 ^B^	− 36.69 ± 3.52 ^B^	30.50 ± 13.75 ^B^
(3) Vitis TP and MW	0.31 ± 0.03^a,A^	0.62 ± 0.03^b,A^	0.48 ± 0.02^c,C^	44.80 ± 9.22 ^C^	− 22.08 ± 4.03 ^C^	56.84 ± 19.22 ^C^
(4) Oral B TP and MW	0.31 ± 0.04^a,A^	0.61 ± 0.03^b,A^	0.41 ± 0.03^c,B^	68.41 ± 11.06 ^B^	− 33.78 ± 5.30 ^B^	33.52 ± 19.31 ^B^
(5) MI paste ONE TP and Caphosol MW	0.29 ± 0.04^a,A^	0.61 ± 0.03^b,A^	0.46 ± 0.01^c,C^	47.83 ± 8.47 ^C^	− 24.28 ± 4.02 ^C^	57.32 ± 19.07 ^C^

*TP* toothpaste, *MW* mouthwash.

In each row, means with the same lowercase letter are not significantly different (Intragroup analysis).

In each column, means with the same capital letter are not significantly different (intergroup analysis).

As presented in Table 4, the surface loss of the remineralized dentin compared to eroded and sound dentin was also calculated to determine the effect of toothpaste and mouthwash of each group more precisely. All interventions could significantly prevent surface loss after the erosive challenge compared to the negative control (p < 0.001 all). The lowest surface loss values were observed in group 2 (Elmex) (− 36.69 ± 3.52) and group 4 (Oral B) (− 33.78 ± 5.30), followed by group 5 (MI Paste ONE/Caphosol) (− 24.28 ± 4.02) and group 3 (Vitis) (− 22.08 ± 4.03), respectively. The differences in the %Surface loss_remineralization-erosion_ between groups 2 and 4 (p = 0.75) and groups 3 and 5 (Vitis and MI Paste ONE/Caphosol) (p = 0.915) were non-significant. Note that the negative values indicate the ability of the toothpastes and mouthwashes used to reduce the roughness caused by the initial acid attack. As shown in Table 4, none of the toothpaste and mouthwash combinations were able to completely restore the dentin surface roughness to the level of sound dentin. In other words, the surface roughness values of remineralized dentin of all groups were higher than the baseline values. The lowest surface loss values were observed in group 2 (Elmex) (30.50 ± 13.75) and group 4 (Oral B) (33.52 ± 19.31), followed by group 3 (Vitis) (56.84 ± 19.22) and group 5 (MI Paste ONE/Caphosol) (57.32 ± 19.07), respectively. The difference in the %Surface loss_remineralization-baseline_ between groups 2 and 4 (Elmex and Oral B) (p = 0.996) and groups 3 and 5 (Vitis and MI Paste ONE/Caphosol) (p = 1.000) was not significant.

### Surface topographic evaluation

**Figure 1:**
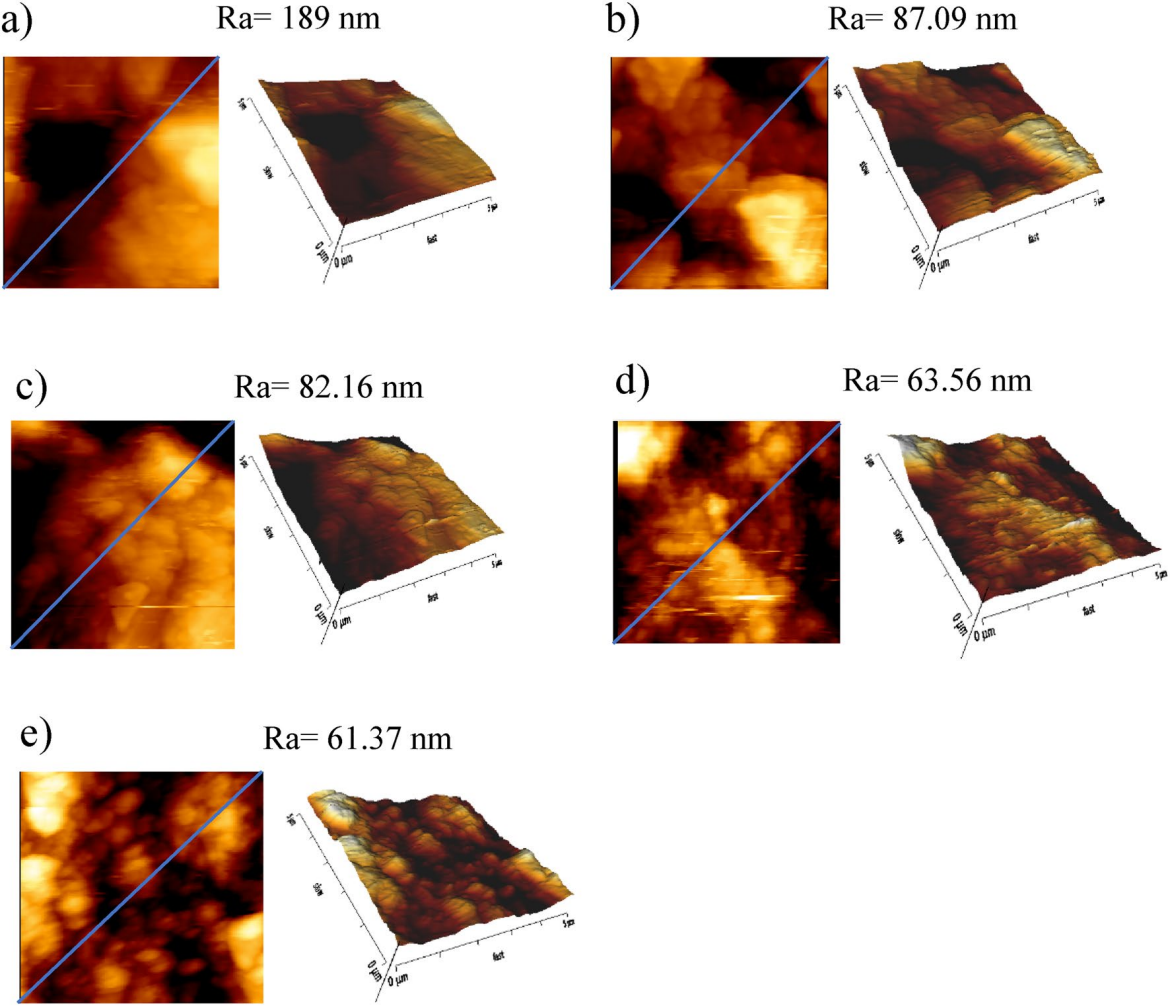
The dentin topographic changes after (**a**) erosion, (**b**) Vitis toothpaste and mouthwash, (**c**) MI paste ONE toothpaste and Caphosol mouthwash, (**d**) Oral B toothpaste and mouthwash, (**e**) Elmex toothpaste and mouthwash.

The AFM findings were consistent with the data obtained from the profilometry. The results are presented as mean ± SD. Erosion led to the increased surface roughness of approximately 187.32 ± 1.21 nm. Group 3 (Vitis) and group 5 (MI Paste ONE toothpaste/Caphosol mouthwash) recovered the surface roughness to 87.25 ± 1.10 nm and 83.50 ± 1.37 nm, respectively. Group 4 (Oral B) exhibited a reduced surface roughness value of 64.03 ± 1.09 nm. Group 2 (Elmex) showed the best recovery of surface roughness, with the lowest measured surface roughness values (60.77 ± 2.06 nm). Figure 1a–e depicts the dentin topographic changes after erosion, after the use of Vitis, MI Paste ONE/Caphosol, Oral B, and Elmex toothpastes and mouthwashes.

## Discussion

Most of the research papers have focused on the effects of different compounds on preventing dental erosion^22–25^, while fewer studies have investigated the quantitative effects of various compounds on eroded tooth surfaces^26–28^. Hence, the present study aimed to evaluate the remineralization effects of four combinations of toothpastes and mouthwashes on eroded dentin surface. This study design resembles the clinical situation to some extent, as most patients seek treatment after the erosion has occurred. Surface microhardness and roughness evaluations are simple, common, and non-destructive methods in re/demineralization studies related to dental erosion^29–31^. For these assessments, the samples are polished to eliminate inherent surface roughness and create identical surfaces in all dental sections^32^.

The sound and eroded dentin microhardness values of the present study were nearly similar to previous studies^31,33^. AmF, NaF, SnF_2_, SMFP, SnCl_2_, nHA, and CPP-ACP were the active ingredients present in the composition of the toothpastes and mouthwashes of the current study, used for remineralization of the eroded dentin surface. Exposure to these compounds resulted in an increase in the average surface microhardness and a decrease in the surface roughness values, in line with the findings of previous studies^3,30,34^. Elmex erosion protection toothpaste and mouthwash, containing stannous and fluoride ions, were used as the positive control in the present study. These compositions are reported to increase acid resistance, reduce tooth solubility, and prevent the activation of matrix metalloproteinases (MMPs) in the eroded tooth surface^3,35^. Due to differences in histology, anti-erosive compounds are less protective on dentin compared to enamel^36^. The deposition of Ca(SnF_3_)_2_, SnO_2_HPO_4_, and Sn_3_F_3_PO_4_ on the tooth surface with greater acid resistance compared to calcium fluoride makes Elmex erosion protection mouthwash as one the best choices for controlling dental erosion^3,37^. At higher pH levels, stannous ions interfere with lesion remineralization in the co-presence of fluoride ions by minimizing fluoride's effects on mineral redistribution within the lesion. However, during the pH drops, stannous ions are adsorbed to the dissolution sites on enamel crystals and provide acid resistance^38^. This might explain the higher acid resistance in Groups 2 and 4.

In accordance with earlier investigations^11,33^, NaF/SnF_2_-containing toothpaste and mouthwash (Oral B) led to an increase in surface microhardness and a decrease in surface roughness. This finding is attributed to the formation of a calcium fluoride layer, which temporarily protects the tooth surface during the acid challenge^9^. We found a lower remineralization effect in Vitis toothpaste and mouthwash than in Elmex toothpaste and mouthwash, which is similar to Guntermann et al.^16^ and de Melo Alencar et al.^11^. The efficacy and the mechanism of action of the HA particles have been reported to depend on the specific geometry, pore size, and degradation rate of the particles^16^. According to Arnold et al.^39^, Elmex toothpastes demonstrated higher remineralization potential followed by NaF and SMFP formulations, which is explained by different amounts of deposited CaF_2_ on the tooth surface due to different solubility of NaF, SMFP and AmF. Despite NaF, the fluoride release of SMFP is dependent on the hydrolysis step, which results in lower salivary and whole plaque fluoride concentration^40^. The presence of SMFP, with weaker remineralization potential, in Vitis toothpaste and mouthwash might be another explanation for the least recovery of enamel surface microhardness and roughness, as there is no phosphatase in the system to hydrolyze it.

Our results represented better performance of the Elmex and Oral B toothpaste and mouthwash compared to MI Paste ONE toothpaste and Caphosol mouthwash, which is in accordance with Lennon et al.^41^. Because of the presence of various remineralizing agents in the composition of each toothpaste and mouthwash, it does not necessarily mean that fluoride has higher remineralizing effect than CPP-ACP. This finding can be explained in several ways as follows: (a) although the CPP-ACP complex can enhance Ca and P mineral precipitation on the eroded surface, these salts are probably soluble under erosive conditions^42^; (b) the concentration of fluoride ion is lower in MI Paste ONE toothpaste than Elmex and Oral B toothpastes; (c) unlike other mouthwashes in the present study, Caphosol mouthwash does not contain fluoride ions; (d) Elmex and Oral B toothpaste and mouthwash contain stannous ions, which adds to the remineralization potential; and (e) toothpaste pH is an influential factor in remineralization potential. Toothpastes with acidic pH have a greater ability to remineralize tooth surfaces during erosive challenges compared to toothpastes with neutral p H^11,16^. The acidic pH of Elmex erosion protection (pH=4.7) and Oral B (pH=5) groups might partly explain their better performance than MI Paste ONE and Vitis anticaries biorepair toothpastes (pH=7, both).

The AFM findings were in accordance with the surface roughness data. As shown in Fig. 1a, the acid challenge increased the surface roughness and caused peritubular and intertubular dentin loss. This finding was in agreement with the outcomes of Satish et al.^43^. The best recovery of surface roughness was related to Elmex, Oral B, MI Paste ONE, and Vitis group, in descending order (Fig. 1 (b-e)).

After acid exposure, the tooth surface is more susceptible to further mineral loss in the presence of abrasive factors such as toothbrushing and toothpastes. The bristles' stiffness, brushing duration, and force can affect dental erosion^44^. For the present study, the brushing force was 2.5 N, based on Ganss et al.^45^, and the brushing method was performed according to Moretto et al. and Levy et al.^46,47^. This method does not significantly increase the erosive tooth wear due to low frequency (twice a day) and short duration (15 s)^48^. Besides, the 30-min interval between the erosive and abrasive challenges might have minimized the abrasion by close contact of dentin with the remineralizing agents and artificial saliva, as suggested by de Souza et al.^3^.

Despite the valuable insights provided by laboratory investigations for decision-making, these studies have certain limitations. We tried to address some of these limitations by accurately following the manufacturers' instructions, preserving the samples in artificial saliva, and simulating the erosive cycles mimicking the acid attack in the oral cavity. The number and duration of the brushing/rinsing challenge were designed to imitate the routine daily oral care during the abrasive cycles. In addition, the brushing force was constant for all dentin samples, and the interventions were carried out by a single blind researcher to prevent personal error and bias. The comparison of commonly used toothpaste and mouthwash in patients with erosive lesions (Elmex erosion protection) with cost-effective alternative formulations for tooth remineralization is another advantage of this study. The absence of enough studies with the same study design precluded ideal comparisons. Therefore, we compared our results with studies closely related to the present investigation. Future studies with a series of erosive challenges under clinical conditions are highly recommended.

## Conclusion

All toothpaste and mouthwash combinations used in the present study led in an increase in dentin microhardness and a decrease in surface roughness values. Elmex and Oral B toothpastes and mouthwashes demonstrated the most significant impact on recovering the surface microhardness and roughness values, followed by MI Paste ONE toothpaste/Caphosol mouthwash and Vitis toothpaste and mouthwash.

## Data Availability

The datasets generated and/or analyzed during the current study are available from the corresponding author upon reasonable request.
